# Modelling the Impact of Vaccination and Other Intervention Strategies on Asymptomatic and Symptomatic Tuberculosis Transmission and Control in Thailand

**DOI:** 10.3390/vaccines13080868

**Published:** 2025-08-15

**Authors:** Md Abdul Kuddus, Sazia Khatun Tithi, Thitiya Theparod

**Affiliations:** 1 Department of Mathematics, University of Rajshahi, Rajshahi 6205, Bangladesh; makuddus.math@ru.ac.bd (M.A.K.); s2512521502@ru.ac.bd (S.K.T.); 2 Department of Mathematics, Mahasarakham University, Maha Sarakham 44150, Thailand

**Keywords:** tuberculosis, mathematical modelling, sensitivity and scenarios analysis, vaccination, Thailand

## Abstract

**Background:** Tuberculosis (TB) remains a major global health challenge, including in Thailand, where both asymptomatic and symptomatic cases sustain transmission. The disease burden increases treatment complexity and mortality, requiring integrated care and coordinated policies. **Methods:** We developed a deterministic compartmental model to examine the transmission dynamics of TB in Thailand, incorporating both latent and active stages of infection, as well as vaccination coverage. The model was calibrated using national TB incidence data, and sensitivity analysis revealed that the TB transmission rate was the most influential parameter affecting the basic reproduction number (*R*_0_). We evaluated the impact of several intervention strategies, including increased treatment coverage for latent and active TB infections and improved vaccination rates. **Results:** Our analysis indicates that among the single interventions, scaling up effective treatment for latent TB infections produced the greatest reduction in asymptomatic and symptomatic cases, while enhanced treatment for active TB cases was second most effective for reducing both asymptomatic and symptomatic cases. Importantly, our results indicate that combining multiple interventions yields significantly greater reductions in overall TB incidence than any single approach alone. Our findings suggest that a modest investment in integrated TB control can substantially reduce TB transmission and disease burden in Thailand. However, complete eradication of TB would require a comprehensive and sustained investment to achieve near-universal coverage of both preventive and curative strategies. **Conclusions:** TB remains a significant public health threat in Thailand. Targeted interventions and integrated strategies are key to reducing disease burden and improving treatment outcomes.

## 1. Introduction

Tuberculosis (TB) is a potentially fatal and life-threatening infectious disease caused by *Mycobacterium tuberculosis (MTB).* Although pulmonary TB mostly affects the lungs, extrapulmonary TB can also affect other organs. Transmission occurs via the inhalation of airborne droplets released by individuals with active TB, posing a significant public health threat, particularly in overcrowded and resource-limited settings. There are two primary types of TB: latent TB, in which a person is infected but shows no symptoms, and active TB, which is typified by clinical signs like fever, weight loss, and a persistent cough. Although latent cases do not present immediate clinical signs, they carry the risk of progressing to active disease and may contribute to ongoing transmission [1].

Globally, TB remains one of the top ten causes of death from infectious disease. According to the World Health Organization, an estimated 10.6 million people contracted TB in 2022, resulting in approximately 1.3 million deaths [2]. Despite improvements in diagnosis and treatment, TB continues to burden low- and middle-income countries due to factors such as late detection, drug resistance, and asymptomatic transmission [3].

Thailand is classified among the 30 high TB burden countries by the WHO, with an estimated incidence of over 150 cases per 100,000 population annually [4]. Thailand presents a unique and complex context for TB control, shaped by several intersecting demographic, social, and health system factors. One of the most critical challenges is the country’s rapidly aging population, which increases the prevalence of latent TB infection (LTBI). LTBI occurs when a person is infected with *MTB* but does not have active TB disease. The bacteria remain dormant (inactive) in the body and cause no symptoms. However, LTBI is clinically important because it represents a reservoir from which active TB disease can emerge [2] and the risk of reactivation due to age-related immunosenescence and comorbidities. Additionally, Thailand experiences significant internal migration, particularly from rural to urban areas, often leading to overcrowded living conditions and limited healthcare continuity, both of which contribute to increased TB transmission risks. Cross-border migration, especially along the western and northeastern borders, further complicates disease surveillance and treatment continuity, as many migrants may have limited access to healthcare services or face legal and language barriers. These population movements heighten the risk of TB spread, and challenge national efforts toward disease elimination.

Moreover, there are notable regional disparities in healthcare infrastructure and resource allocation, which result in uneven access to TB diagnosis, treatment, and follow-up care across provinces. Rural and underserved areas often struggle with healthcare workforce shortages and inadequate laboratory capacity, delaying detection and contributing to poorer treatment outcomes. Despite significant advances in TB control, including Directly Observed Treatment Shortcourse (DOTS) implementation and Bacillus Calmette–Guerin (BCG) vaccination programs, the persistence of asymptomatic carriers and undetected latent TB infections remains a major obstacle to achieving TB elimination [2,5]. These silent carriers may unknowingly spread the disease, complicating control measures and increasing the risk of outbreaks, particularly in urban and marginalized communities [6]. These complexities necessitate a context-specific modeling approach to evaluate the potential impact of various intervention strategies and inform evidence-based public health policies tailored to Thailand’s epidemiological and socio-demographic landscape.

Vaccination, along with other intervention strategies such as the treatment of latent and active TB, plays a critical role in reducing TB incidence and mortality. Emerging TB vaccines, when integrated with improved case detection, preventive therapy, and strategies to enhance treatment adherence, provide a comprehensive approach to controlling both asymptomatic and symptomatic transmission [7,8,9]. Collectively, these interventions not only mitigate the overall disease burden but also interrupt transmission chains within the population, contributing to more effective TB control.

Comprehensive knowledge of vaccine and treatment approaches is necessary to effectively combat TB. Mathematical modeling can provide critical insights into how different levels of investment and intervention coverage can influence TB incidence and prevalence in both short- and long-term timeframes. Previous modeling studies have estimated the importance of integrated, multi-component strategies for reductions in disease burden compared to single-policy approaches [3,5,9,10], but have not explicitly investigated the impact of vaccination and treatment on the dynamics of asymptomatic and symptomatic TB in Thailand.

This study presents a robust mathematical modeling framework designed to evaluate the impact of vaccination coverage and targeted public health interventions on the transmission dynamics of both asymptomatic and symptomatic TB in Thailand. The model integrates key epidemiological features of TB, including latent infection, asymptomatic carriers, progression to active disease, vaccination, and treatment interventions. By simulating a range of control scenarios over a 15-year horizon, the framework facilitates an extensive evaluation of the effectiveness of single and combined strategies, such as increased vaccination coverage, improved treatment for latent and active TB infections, and their respective programmatic scales.

## 2. Methods and Materials

In this study, we developed a compartmental deterministic model to characterize the transmission dynamics and control strategies of TB in Thailand. The model explicitly incorporates both asymptomatic and symptomatic transmission pathways, as well as the impact of vaccination on disease progression and control.

The total population is divided into six mutually exclusive compartments: susceptible individuals S(t), vaccinated individuals V(t), latent individuals L(t), asymptomatic infectious individuals A(t), symptomatic infectious individuals I(t), and recovered individuals R(t). At the given time t, the total population N(t) is expressed as:

(1) $$N\left(t\right)=\;S(t)+V(t)+L(t)+A(t)+I(t)+R(t)$$

The model assumes that the total population size remains constant over time, with the inflow of new births balancing natural deaths. Natural death refers to the baseline mortality in the population that occurs independently of the disease under study. In our proposed model, it is typically represented by a constant per capita death rate, denoted by μ, and affects all compartments equally. This population assumption simplifies the demographic dynamics, allowing a focused investigation of TB transmission and intervention effects within a stable population framework. By incorporating both vaccination strategies and distinct infectious states, the model aims to provide deeper insights into the relative contributions of asymptomatic and symptomatic transmission to the overall TB burden and to evaluate the potential impact of vaccination and treatment on disease control in the Thai population.

In the model, susceptible individuals enter the population at a constant recruitment rate denoted by μN, which represents births or the influx of new individuals into the system, and all compartments are subject to natural mortality at the rate μ. Susceptible individuals face the risk of infection through effective contact with either symptomatic infectious individuals I(t) or asymptomatic infectious individuals A(t).

The force of infection, which defines the per capita rate at which susceptible individuals acquire infection, is given by:

$$\lambda \left(t\right)=\beta \left[I\left(t\right)+\eta A\left(t\right)\right]$$

where β is the transmission rate of TB and η∈[0, 1] represents the modulation parameter that adjusts the relative infectiousness of asymptomatic individuals compared to symptomatic ones. A lower value of η indicates that asymptomatic individuals are less infectious, while η=1 would imply they are equally infectious as symptomatic cases.

Following exposure, susceptible individuals who become infected transition to the latent compartment L(t), which represents individuals who have been infected but are not yet capable of transmitting the disease. This latent stage is critical in TB epidemiology, as it captures the delay between initial infection and the onset of infectiousness, allowing for the possibility of either progression to active disease or maintenance in a non-infectious state. Latently infected individuals progress to either asymptomatic or symptomatic TB. Specifically, a fraction ρα of latent individuals develops symptomatic infection I(t), while the remaining (1−ρ)α develops asymptomatic infection A(t), where α is the overall activation rate from latency. Again, latent individuals may recover at a rate ψ and move to R(t). Asymptomatic individuals may naturally recover at a rate $\delta_{1}$, or progress to symptomatic TB at a rate κ. Symptomatic individuals may recover at a treatment-induced rate τ and naturally recover at a rate $\delta_{2}$, or die from TB-induced complications at a rate $\mu_{I}$. Additionally, symptomatic individuals I(t) move to A(t) at a rate ω.

In this model, vaccination is represented as a single-dose preventive strategy aimed at reducing susceptibility to TB infection. Susceptible individuals are vaccinated at a rate $\sigma_{1}$ and subsequently enter the vaccinated compartment V(t). We assume homogeneous vaccination among the susceptible population, without distinguishing between prevaccinated and unvaccinated individuals. While this simplifies the system, it may overlook behavioral heterogeneity in vaccine uptake. Introducing separate compartments for these subgroups would enhance realism but is unlikely to alter the qualitative dynamics significantly, as the key distinction lies in the infection rate rather than in structural model flows. However, vaccination does not confer lifelong immunity. Vaccinated individuals are assumed to lose vaccine-induced protection over time and transition to the recovered compartment R(t) at a rate γ, reflecting waning immunity or partial protection mechanisms. Both vaccinated and recovered individuals are assumed to have temporary immunity. Due to immune decline, vaccinated individuals may return to the susceptible class at a rate $\sigma_{2}$, while recovered individuals lose natural immunity and re-enter the susceptible pool at a rate ϕ.

**Figure 1:**
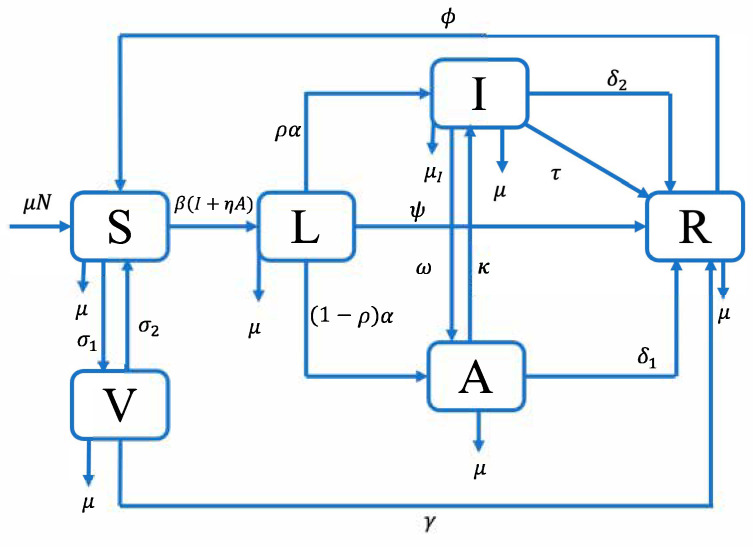
Schematic diagram of the TB transmission model with vaccination and intervention strategies in Thailand.

The resulting system of nonlinear ordinary differential equations is used to explore the dynamic behavior of the TB epidemic under different levels of vaccination, treatment, and detection. The structure of the model is represented by the flow diagram shown in Figure 1, which illustrates the transmission and control pathways of TB. The transmission dynamics of TB are described by the following system of nonlinear ordinary differential equations derived from the formulated deterministic model.

(2) $$\left\{\begin{array}{c} \frac{dS}{dt}=\mu N-\beta S\left(I+\eta A\right)-S\left(\sigma_{1}+\mu \right)+\sigma_{2}V+\phi R\;\;\;\;\;\;\;\;\;\;\;\;\\ \frac{dV}{dt}=\sigma_{1}S-{(\sigma }_{2}+\mu +\gamma )V\;\;\;\;\;\;\;\;\;\;\;\;\;\;\;\;\;\;\;\;\;\;\;\;\;\;\;\;\;\;\;\;\;\;\;\;\;\;\;\;\;\;\;\;\;\;\;\;\;\;\;\;\\ \frac{dL}{dt}=\beta S\left(I+\eta A\right)-L\left(\alpha +\mu +\psi \right)\;\;\;\;\;\;\;\;\;\;\;\;\;\;\;\;\;\;\;\;\;\;\;\;\;\;\;\;\;\;\;\;\;\;\;\;\;\;\;\;\;\\ \frac{dA}{dt}=\left(1-\rho \right)\alpha L+\omega I-A\left(\kappa +\mu +\delta_{1}\right)\;\;\;\;\;\;\;\;\;\;\;\;\;\;\;\;\;\;\;\;\;\;\;\;\;\;\;\;\;\;\\ \frac{dI}{dt}=\rho \alpha L-I\left(\mu +\omega +\tau +\mu_{I}+\delta_{2}\right)+\kappa A.\;\;\;\;\;\;\;\;\;\;\;\;\;\;\;\;\;\;\;\;\;\;\;\\ \frac{dR}{dt}=\gamma V+\delta_{1}A-R\left(\mu +\phi \right)+\psi L+\tau I+\delta_{2}I.\;\;\;\;\;\;\;\;\;\;\;\;\;\;\;\;\; \end{array}\right.$$

With the following positive initial conditions,

(3) $$S\left(0\right)=S_{0}\geq 0,\;V\left(0\right)=V_{0}\geq 0,\;L\left(0\right)=L_{0}\geq 0,\;A\left(0\right)=A_{0}\geq 0,\;I\left(0\right)=I_{0}\geq 0,R\left(0\right)=R_{0}\geq 0$$

the boundedness of the solution (for all t≥0) of the proposed model (2) with the initial conditions (3) can be easily performed.

### 2.1. Boundedness

In this section, we analyze the model (2) to determine the biological feasible solution set. The following theorem assures that the solutions of the system are bounded in the set with the non-negative conditions.

**Theorem** **1**  *The feasible solution set of the system (2) subjected to the initial conditions (3) which initiate in* $R_{+}^{6}$ *are uniformly bounded in Γ, where* $\Gamma =\left\{\left(S,\;V,\;L,\;A,\;I,\;R\right)\in \;R_{+}^{6}:S+V+L+A+I+R=N\right\}$ *is the positively invariant region.*

**Proof.**  Using the non-negative initial conditions of (3) in the system (2), it is observed that each of the dynamical variables remains non-negative. So, adding each of the equations of the system (2), we obtain the total population size, N(t), which satisfies in the absence of death due to TB, then we get

$$\frac{dN}{dt}=\frac{dS}{dt}+\frac{dV}{dt}+\frac{dL}{dt}+\frac{dA}{dt}+\frac{dI}{dt}+\frac{dR}{dt}=0$$

Integrating the above equation, we have N(t) = constant. □

Accordingly, given the assumption of a constant total population size, it follows that all feasible solutions of the dynamical variables S, V, L, A, I, R are bounded within a positively invariant region.

### 2.2. Basic Reproduction Number

The basic reproduction number, commonly denoted as ${\;R}_{0}$, is a foundational metric in the study of infectious disease dynamics. It quantifies the average number of secondary infections generated by one primary infectious individual in a completely susceptible population. This parameter serves as a threshold indicator that helps determine whether an infectious disease is likely to spread or die out in a given population. When $R_{0}>1$, each infected person is expected to infect more than one individual, leading to potential epidemic expansion and sustained transmission within the population. This scenario signals a public health concern and necessitates immediate intervention to curb disease spread. Conversely, if $R_{0}<1$, the infection fails to replace itself over time, resulting in a declining trajectory of disease cases. Under such conditions, the pathogen is likely to fade out from the population. In the special case where $R_{0}=1$, the infection maintains a steady-state transmission, implying that the number of new cases remains constant over time without exponential growth or decline.

The model consists of three uninfected states (S,  V, and  R) and three infected states (L, I, and A). The model has six distinct states in such a scenario, while the overall population stays constant. At the free steady diseases $L^{0}=I^{0}=A^{0}=0$. Therefore, $S^{0}\;=\frac{\mu N\left(\sigma_{2}+\mu +\gamma \right)\left(\mu +\phi \right)}{\left(\sigma_{1}+\mu \right)\left(\sigma_{2}+\mu +\gamma \right)\left(\mu +\phi \right)-\sigma_{1}\sigma_{2}\left(\mu +\phi \right)-\phi \gamma \sigma_{1}}$, $V^{0}\;=\;\frac{\mu N\sigma_{1}\left(\mu +\phi \right)}{\left(\sigma_{1}+\mu \right)\left(\sigma_{2}+\mu +\gamma \right)\left(\mu +\phi \right)-\sigma_{1}\sigma_{2}\left(\mu +\phi \right)-\phi \gamma \sigma_{1}}$, and $R^{0}=\frac{\gamma \mu N\sigma_{1}}{\left(\sigma_{1}+\mu \right)\left(\sigma_{2}+\mu +\gamma \right)\left(\mu +\phi \right)-\sigma_{1}\sigma_{2}\left(\mu +\phi \right)-\phi \gamma \sigma_{1}}$. Hence, for (L, I, and A), we have the following system:

(4) $$\left\{\begin{array}{c} \frac{dL}{dt}=\beta S\left(I+\eta A\right)-L\left(\rho \alpha +\left(1-\rho \right)\alpha +\mu +\psi \right)\\ \frac{dI}{dt}=\rho \alpha L-I\left(\mu +\omega +\tau +\mu_{I}+\delta_{2}\right)+\kappa A\;\;\;\;\;\;\;\;\;\\ \frac{dA}{dt}=\left(1-\rho \right)\alpha L+\omega I-A\left(\kappa +\mu +\delta_{1}\right)\;\;\;\;\;\;\;\;\;\;\;\;\;\; \end{array}\right.$$

Our framework ODE (4) is the subsystem for infection. It only defines the creation of new infections and modifications to the conditions of previously existing infections. Suppose we set $X^{T}=(L,\;I,\;A)$ where T stands for transpose. We now want to express the subsystem as follows:

(5) $$\;\;\;{\dot{X}}^{.}=\;(F+V)\;X\;\;$$

We found the two matrices from Equation (4) by polarizing the transmission events from each other proceedings. When we designate the affected states as having indices i, j and i, j ∈ 1, 2, 3, the rate at which an individual state j generates individuals in the system’s infected state i is known as the entry $V_{ij}$. Thus, in the case of subsystem (4), we get

(6) $$F=\left[\begin{array}{ccc} 0 & \beta S^{0} & \beta \eta S^{0}\\ 0 & 0 & 0\\ 0 & 0 & 0 \end{array}\right]\mathrm{and}\;V^{-1}=\left[\begin{array}{ccc} \left(\alpha +\mu +\psi \right) & 0 & 0\\ -\rho \alpha & \mu +\omega +\tau +\mu_{I}+\delta_{2} & -\kappa \\ -\left(1-\rho \right)\alpha & -\omega & \kappa +\mu +\delta_{1} \end{array}\right]$$

Here, K is the next-generation matrix [10] with an essential minus sign.

(7) $$K=-FV^{-1}$$

A single infected person can create an average of one new infection, which is represented by the dominating eigenvalue of K in this instance. Thus, the basic reproduction number is

(8) $$R_{0}=\frac{\mu N\alpha \beta \left(\sigma_{2}+\mu +\gamma \right)(\phi +\mu )(\kappa +B\eta +C\rho -\kappa \rho -B\eta \rho +\eta \omega \rho )}{\left[\left(\sigma_{1}+\mu \right)\left(\sigma_{2}+\mu +\gamma \right)\left(\phi +\mu \right)-\sigma_{1}\sigma_{2}\left(\mu +\phi \right)-\phi \gamma \sigma_{1}]A\;(BC-\kappa \omega \right)}$$

where

$$A=(\alpha +\mu +\psi ),\;B=(\mu +\omega +\tau +\mu_{i}+\delta_{2}),\;\mathrm{and}\;C=(\kappa +\mu +\delta_{1}).$$

### 2.3. Model Evaluation

The following section provides a comprehensive overview of the foundational components of the proposed TB transmission dynamics framework (2).

#### Steady-State Analysis: Disease-Free Equilibrium and Endemic Equilibrium

In this section, we present the system properties of two biologically and mathematically meaningful equilibrium states of the proposed model. The first is the disease-free equilibrium (DFE), representing a scenario in which the basic reproduction number $R_{0}$ is less than one. Under this condition, the infection cannot establish itself or persist in the population, and the disease eventually dies out. The second is the endemic equilibrium, which arises when $R_{0}>1$, indicating that the infection can sustain transmission and remain prevalent within the population over time. It is important to highlight that the disease-free equilibrium always exists for system (2), regardless of parameter values, as it corresponds to a state where no individuals are infected making it a fundamental reference point for model behavior. The existence and stability of these equilibria provide critical insight into the potential outcomes of TB dynamics under varying epidemiological conditions.

(9) $$E^{0}=\left(S^{0},\;V^{0},\;{L^{0},\;A}^{0},I^{0},\;R^{0}\right)$$

where $S^{0}=\frac{\mu N\left(\sigma_{2}+\mu +\gamma \right)\left(\phi +\mu \right)}{\left(\sigma_{1}+\mu \right)\left(\sigma_{2}+\mu +\gamma \right)\left(\mu +\phi \right)-\sigma_{1}\sigma_{2}\left(\mu +\phi \right)-\phi \gamma \sigma_{1}},$ $V^{0}=\frac{\mu N\;\sigma_{1}\left(\phi +\mu \right)}{\left(\sigma_{1}+\mu \right)\left(\sigma_{2}+\mu +\gamma \right)\left(\mu +\phi \right)-\sigma_{1}\sigma_{2}\left(\mu +\phi \right)-\phi \gamma \sigma_{1}}$, $L^{0}=0$, $A^{0}=0,\;I^{0}=0$ and $R^{0}=\frac{\gamma \mu N\sigma_{1}}{\left(\sigma_{1}+\mu \right)\left(\sigma_{2}+\mu +\gamma \right)\left(\mu +\phi \right)-\sigma_{1}\sigma_{2}\left(\mu +\phi \right)-\phi \gamma \sigma_{1}}$

We may also determine the diseases-endemic equilibrium from Equation (2).

(10) $$E^{*}=\left(S^{*},\;V^{*},\;{L^{*},\;A}^{*},I^{*},\;R^{*}\right)$$

where

(11) $$\begin{array}{c} S^{*}=\frac{1}{R_{0}}\frac{\mu N(\sigma_{2}+\mu +\gamma )}{\alpha \beta \Theta }\\ V^{*}=\frac{1}{R_{0}}\frac{\mu N\sigma_{1}}{\alpha \beta \Theta }\\ L^{*}=\frac{\mu N}{A}\left(1-\frac{1}{R_{0}}\right)\\ A^{*}=\frac{\mu N}{AC}\left(1-\frac{1}{R_{0}}\right)E\\ I^{*}=\frac{\mu N\alpha }{AB}\left(1-\frac{1}{R_{0}}\right)D\\ R^{*}=\frac{1}{\mu +\phi }\left(\frac{\gamma \sigma_{1}\mu N}{R_{0}\alpha \beta \Theta }+\frac{\delta_{1}\mu N}{AC}\left(1-\frac{1}{R_{0}}\right)E+\frac{(\tau +\delta_{2})\mu N\alpha }{AB}\left(1-\frac{1}{R_{0}}\right)D+\frac{\psi \mu N}{A}\left(1-\frac{1}{R_{0}}\right)\right) \end{array}$$

where A=(α+μ+ψ), $B=(\mu +\omega +\tau +\mu_{i}+\delta_{2})$, $C=(\kappa +\mu +\delta_{1})$,

(12) $$\begin{array}{c} \Theta =\kappa +B\eta +C\rho -\kappa \rho -B\eta \rho +\eta \omega \rho ,\;D=\rho +\frac{\kappa \left(1-\rho \right)}{C}+\frac{\kappa \omega \rho }{BC-\kappa \omega },\;\mathrm{and}\\ E=\left(1-\rho \right)\alpha +\frac{\omega \alpha }{B}\left(\rho +\frac{\kappa \left(1-\rho \right)}{C}+\frac{\kappa \omega \rho }{BC-\kappa \omega }\right)=\left(1-\rho \right)\alpha +\frac{\omega \alpha }{B}D. \end{array}$$

Therefore, Equations (11) and (12) show that the endemic equilibrium $E^{\;*}=\left(S^{*},\;V^{*},\;{L^{*},\;A}^{*},I^{*},\;R^{*}\right)\in D$ (i.e., exists) if $R_{0}>1$.

## 3. Results

### 3.1. Parameter Estimation

**Figure 2:**
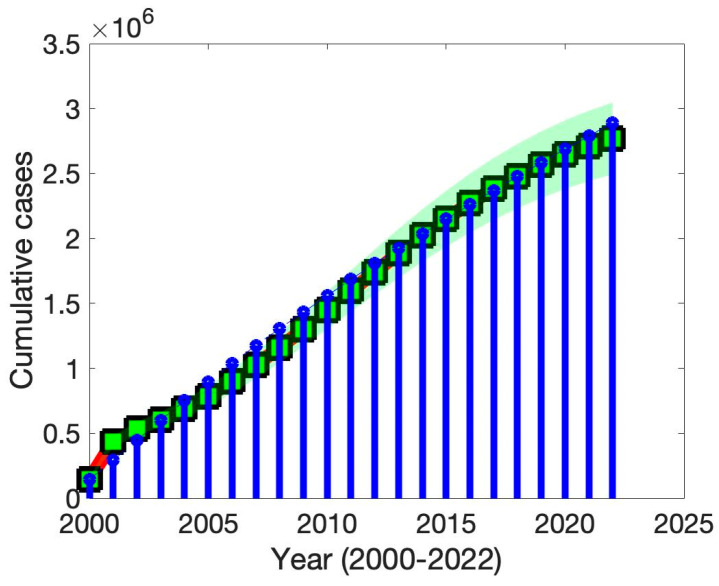
Model fit to reported TB incidence in Thailand with 95% confidence intervals (red solid square curve: model prediction; blue bars: reported cases; green-shaded area: 95% CI).

A defining feature of TB is its mode of transmission, primarily occurring through person-to-person contact. Consequently, the rate of transmission plays a pivotal role in shaping the trajectory of TB epidemics globally. In this section, we estimate the relevant parameter values by analyzing reported TB incidence data from Thailand spanning the period 2000 to 2022. This longitudinal dataset allows us to calibrate our model and evaluate its performance against observed trends. Figure 2 illustrates this calibration process, where the fitted curve is represented by a solid red square line with 95% confidence interval, demarcated by green-shaded boundaries, effectively capturing the general trend in TB incidence over the two-decade period. The corresponding observed case counts are shown as blue bars. The close alignment between the fitted line and empirical data underscores the robustness of our parameter estimation approach. Furthermore, this visual correspondence highlights the model’s capacity to reliably replicate historical transmission patterns, reinforcing the validity of our assumptions and the effectiveness of the underlying modeling framework.

To ensure the accuracy and reliability of our TB transmission model, we determined key epidemiological parameters by fitting the model to the observed TB case data from Thailand. This fitting process was conducted using a robust parameter estimation technique—the least-squares method—which is widely recognized for minimizing the discrepancy between observed data and model predictions. This approach enabled us to identify parameter values that provided the best fit to the observed data while preserving biological plausibility. Specifically, the least-squares approach was applied to our proposed model to reduce the total squared error between the predicted values $P\left(t,x\right)$ and the actual reported TB incidence $Q_{actual}$. The objective function ${\left(P\left(t,\;x\right)-Q_{actual}\right)}^{2}$ quantifies the difference between the model output and real-world data over time, where x represents the set of estimated parameters and t denotes time. This method ensures that the resulting model closely reflects the actual dynamics of TB infection in Thailand.

Through this procedure, we estimated several critical parameters: the transmission rate $\left(\beta \right)=\;2.8\times 10^{-7}(95\%\;CI\;2.3\times 10^{-7},\;3.3\times 10^{-7})$, progression rate $\left(\alpha \right)=0.26\;(95\%\;CI\;0.209,\;0.311)$, relative infection of carriers $\left(\eta \right)=0.0006(95\%CI\;0.00048,0.00072)$, and vaccination rate $\left(\sigma_{1}\right)=0.90(95\%\;CI\;0.724,\;1.076)$. These values were chosen to optimize the model’s predictive accuracy. The estimated transmission rate is closure with values found in earlier compartmental models where the population is scaled [11]. The progression rate from latent to active TB aligns well with biological estimates that latent TB activates [11]. However, the relative infectiousness of carriers is lower than reported estimates [11], which may reflect model assumptions. The vaccination rate corresponds closely with BCG coverage reported by the WHO for the region in question [2].

Additional parameter values were sourced from established peer-reviewed literature and are comprehensively listed in Table 1. This methodological framework not only strengthens the model’s predictive capabilities but also enhances its practical relevance, enabling public health authorities to make informed decisions regarding TB prevention, control, and vaccination strategies tailored to the Thai context.

### 3.2. Sensitivity Analysis

Sensitivity analysis is a critical tool to identify the most influential parameters affecting model outcomes [15,16,17]. In this study, we conduct a sensitivity analysis to determine which parameters have the greatest impact on the model outputs. This process highlights the key drivers of disease dynamics and offers valuable insights for effective outbreak control [18,19,20]. We employ Latin Hypercube Sampling (LHS), a robust and efficient sampling-based technique, combined with Partial Rank Correlation Coefficient (PRCC) analysis, a global sensitivity method [20]. PRCC specifically assesses the monotonic relationships between input parameters and model outputs by analyzing residuals from linear regression models. This residual-based approach is particularly well-suited for nonlinear systems, allowing for a more reliable evaluation of parameter influence.

**Figure 3:**
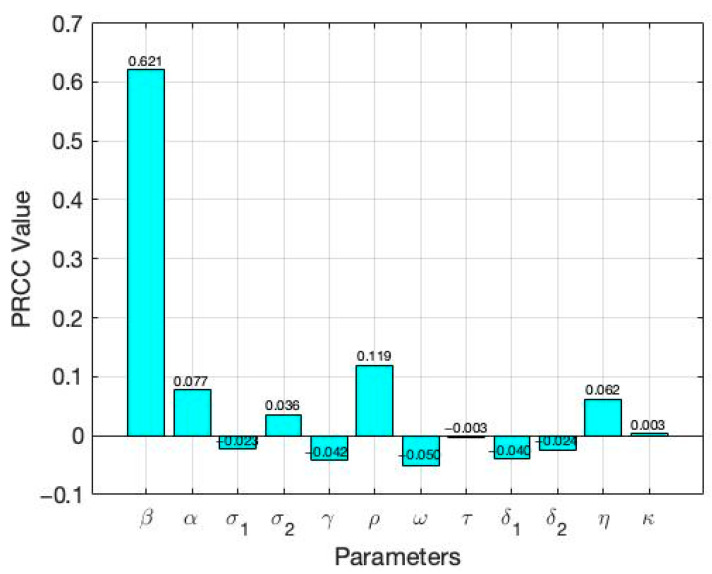
PRCC values showing the influence of key epidemiological parameters on  $R_{0}$.

**Figure 4:**
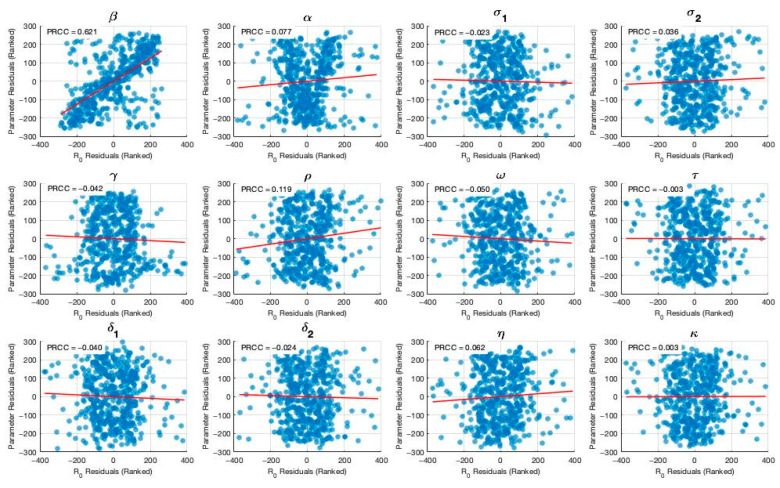
Partial residual plots illustrating the impact of each parameter on $R_{0}$ while controlling for others; red line shows direction and strength. Derived from 400 Monte Carlo simulations Latin Hypercube Sampling.

Figure 3 and Figure 4 present the results of the sensitivity analysis of the basic reproduction number (R_0_) using Latin Hypercube Sampling (LHS) and Partial Rank Correlation Coefficients (PRCC). This analysis evaluates the impact of key epidemiological parameters on $R_{0}$.

The PRCC results were further visualized using a bar plot (Figure 3). Each parameter was ranked based on their relative impact on $R_{0}.$ The results of these analysis indicate that the transmission rate (β) exhibits the highest PRCC value (+0.621), indicating a strong positive influence on $R_{0}$. Two primary visual outputs were generated: the PRCC bar plot (Figure 3) and the residuals plot (Figure 4). The residuals plot in Figure 4 illustrates the partial dependency between each parameter and $R_{0}$ while accounting for the correlations with other variables. Each subplot displays the correlation between the residuals of the ranked input parameter (*y*-axis) and the residuals of the ranked $R_{0}$ values (*x*-axis). The red regression line in each subplot indicates the strength and direction of the partial correlation, where the slope reflects whether the parameter positively or negatively influences $R_{0}$. Parameters with positive slopes—such as $\beta ,\;\alpha ,\;\sigma_{2},\;\rho ,\;\eta$, and κ—are identified as transmission-enhancing factors, while those with negative slopes—such as $\sigma_{1},\gamma$,$\omega ,\;\tau ,\;\delta_{1}$, and $\delta_{2}$—act as transmission-suppressing factors. Negative residuals for $R_{0}$ indicate that the actual value is lower than expected after adjusting for other parameters, suggesting a transmission-suppressing effect. This pattern, observed for parameters such as $\sigma_{1}$ and γ, supports their negative PRCC values and confirms their role in reducing TB transmission. Notably, the transmission rate (β) exhibits the highest PRCC value, indicating a strong positive influence on $R_{0}$. These results strengthen the robustness of our conclusions in two key ways. First, PRCC analysis consistently identified the transmission rate (*β*) as the primary driver of $R_{0}$, aligning with well-established epidemiological evidence and confirming the model’s sensitivity to biologically meaningful parameters. Second, while changes in parameter values influenced the magnitude of $R_{0}$, the relative ranking and effectiveness of interventions remained stable. This indicates that the model’s policy-relevant insights—such as the benefits of vaccination and enhanced treatment—are qualitatively robust, even under parameter uncertainty.

### 3.3. Scenario Analysis

In this section, we developed four potential intervention scenarios to examine the dynamics of asymptomatic and symptomatic TB in Thailand. These scenarios are detailed in Table 2 and Table 3. Thailand’s Strategic Plan for TB Elimination focuses on reducing TB incidence and mortality through early detection, timely and effective treatment, prevention, and strong surveillance. Key strategies include expanding access to rapid diagnostics, improving treatment adherence, providing preventive therapy for high-risk groups, and enhancing infection control measures. The plan emphasizes community engagement, reducing stigma, strengthening health systems, and promoting research and innovation. Aligned with the WHO End TB Strategy, Thailand aims to eliminate TB as a public health threat by 2035.

Scenario 1 represents a continuation of the programmatic conditions projected over the period from 2024 to 2039. Within this framework, we evaluated four distinct single-intervention strategies, each applied independently to assess their individual impact. These interventions included (1) enhancing active TB treatment coverage, (2) improving preventive therapy, targeting individuals identified as having latent TB infection through screening or diagnostic tools, (3) increasing the reporting rate of symptomatic TB cases, and (4) raising the vaccination rate in Thailand. In this context, enhanced active TB treatment refers to improvements in the efficacy, formulation, or delivery of first-line anti-tuberculosis drugs such as isoniazid and rifampicin. Specifically, it assumes increased treatment success rates through measures such as directly observed therapy (DOT) and community-based support programs. Vaccination coverage refers to the proportion or percentage of population that has received a vaccine within a defined period. Each intervention was implemented in isolation and compared against the baseline scenario (see Table 2) to evaluate its effect on asymptomatic and symptomatic TB cases. In this scenario, the active TB treatment and preventive therapy were increased from 0.50 (50%) and 0.17 (17%) in the baseline to 1.00 (100%) for both parameters. Similarly, the symptomatic TB case-reporting rate and vaccination coverage were elevated from 0.30 (30%) and 0.90 (90%) to 1.00 (100%), respectively.

**Figure 5:**
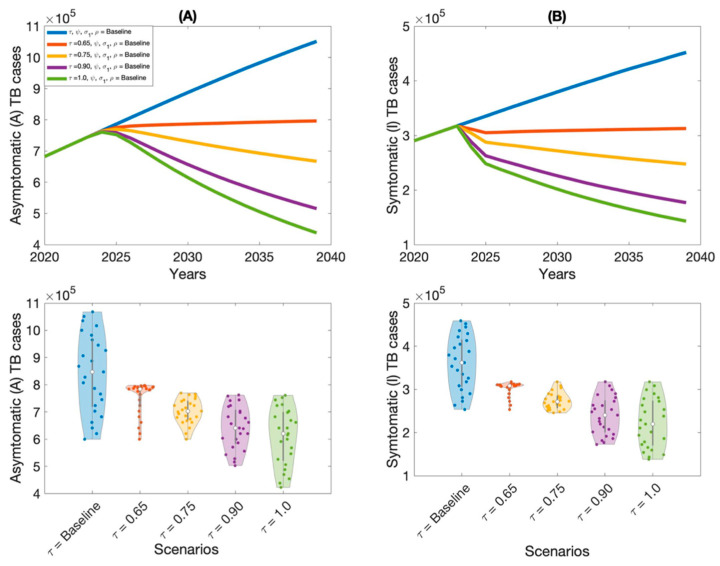
Impact of increasing active TB treatment rate (τ) on asymptomatic TB cases (**A**) and symptomatic TB cases (**B**) in Thailand.

**Figure 6:**
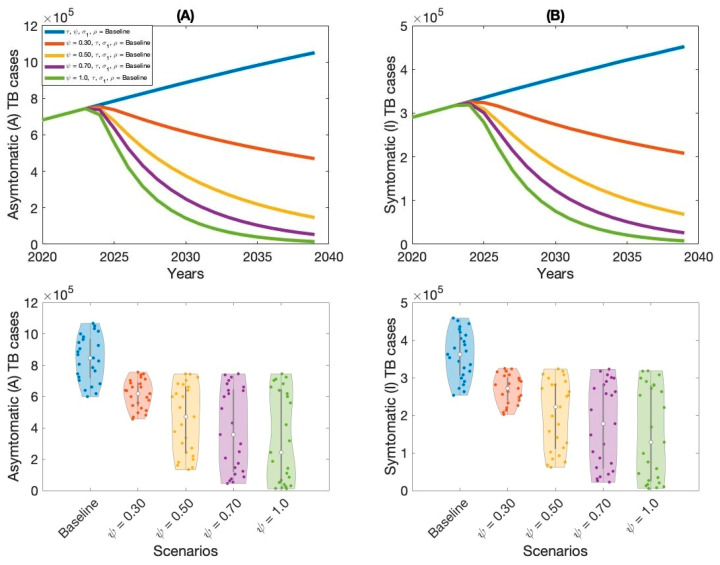
Impact of increasing preventive therapy rate (ψ) on asymptomatic TB cases (**A**), symptomatic TB cases (**B**) in Thailand.

**Figure 7:**
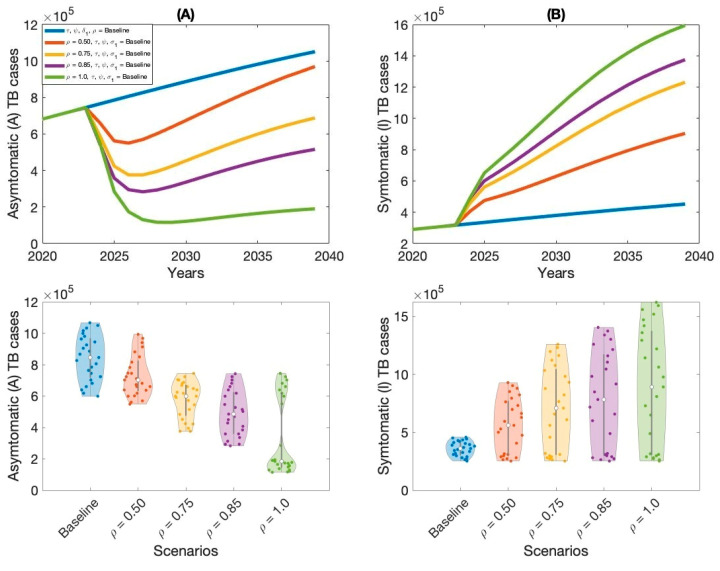
Impact of increasing symptomatic TB reporting rate (ρ) on asymptomatic TB cases (**A**) and symptomatic TB cases (**B**) in Thailand.

**Figure 8:**
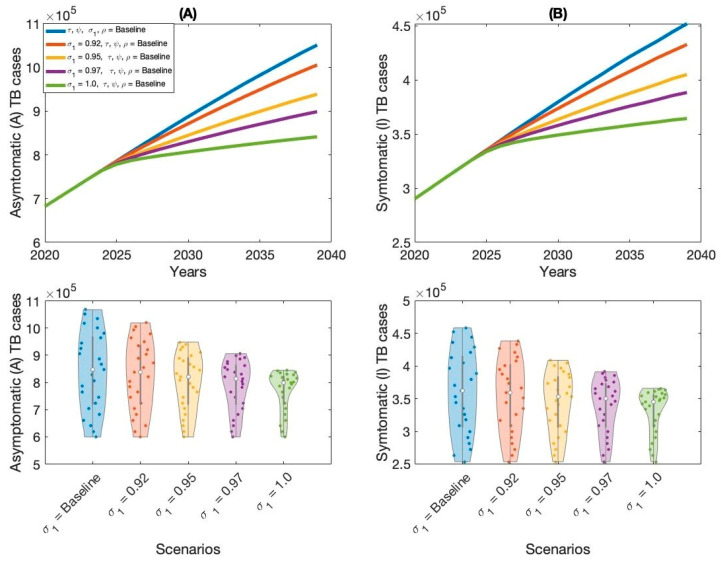
Impact of increasing vaccination rate $\left(\sigma_{1}\right)$ on asymptomatic TB cases (**A**) and symptomatic TB cases (**B**) in Thailand.

The outcomes of Scenario 1 are presented in Figure 5, Figure 6, Figure 7 and Figure 8 and Table 2. This scenario assesses the impact of four individual intervention strategies designed to reduce the TB burden in Thailand. Among these, the strategy focused on preventive therapy proved to be the most effective in reducing overall TB cases, as shown in Figure 6A,B and Table 2. This highlights the critical role of targeting the latent reservoir to prevent progression to active TB disease. We also generated violin plots to visualize the distribution, central tendency, and variability of each strategy. Each violin plot shows the distribution of strategy outcomes, with the width indicating frequency at different values. The median marker reflects the central outcome, the box shows the interquartile range, and individual points reveal variability. This allows comparison of performance, consistency, and uncertainty across strategies. Additionally, the results indicate that increasing treatment coverage for individuals with active TB also leads to substantial reductions in case numbers, as shown in Figure 5A,B and Table 2. Compared to other strategies—such as expanding vaccination coverage or improving the symptomatic case-reporting rate—enhanced active TB treatment demonstrates greater effectiveness in controlling disease transmission. Overall, these findings suggest that preventive therapy remains the most effective single-intervention strategy, with active TB treatment serving as a strong alternative for reducing TB incidence in Thailand.

**Figure 9:**
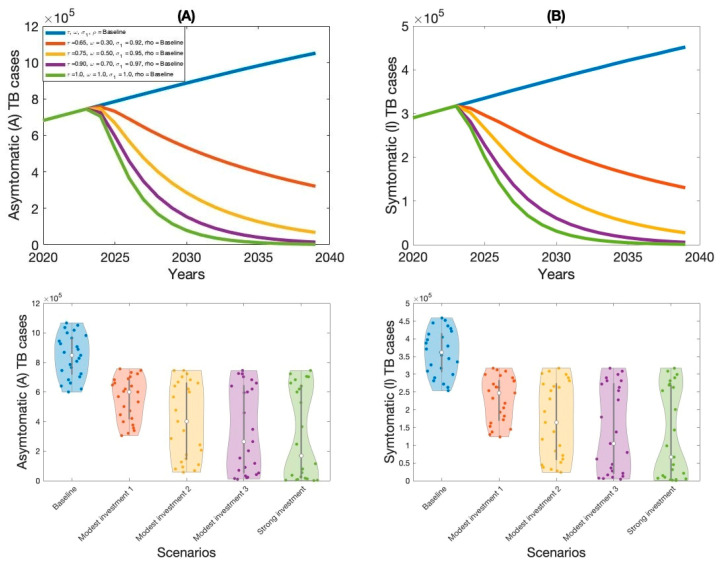
Impact of multiple interventions on asymptomatic TB cases (**A**) and symptomatic TB cases (**B**) in Thailand.

The baseline control strategy incorporates a combination of three key interventions at their existing implementation levels. These include active TB treatment coverage at 50% (0.5), preventive therapy at 17% (0.17), and vaccination coverage at 90% (0.90), as shown in Table 3 and Figure 9. While reflecting current TB control efforts in Thailand, simulations indicate that TB incidence is projected to increase under these conditions. This indicates that the existing control measures are insufficient to suppress the transmission dynamics of the disease. Therefore, a more aggressive or optimized combination of interventions may be necessary to reverse the upward trend and achieve significant reductions in TB prevalence.

Strategy 1 represents a targeted enhancement of three key intervention parameters compared to the baseline approach. Specifically, it increases active TB treatment coverage from 50% (0.50) to 65% (0.65), preventive therapy from 17% (0.17) to 30% (0.30), and vaccination coverage from 90% (0.90) to 92% (0.92) (refer to Table 3 and Figure 9). Simulation results suggested that this modest escalation in intervention efforts leads to a significant reduction in both symptomatic and asymptomatic TB cases across the population. Compared to the baseline scenario, Strategy 1 demonstrates a clear improvement in mitigating disease burden. The decline in TB prevalence underscores the importance of even moderate increases in treatment and vaccination efforts.

Strategy 2 involves a combination of the three key interventions, with coverage increased from baseline levels to 70% (0.70) for active TB treatment, 50% (0.50) for preventive therapy, and 95% (0.95) for vaccination, respectively. The simulation results presented in Table 3 and Figure 9 clearly show that this strategy outperforms Strategy 1 by reducing both symptomatic and asymptomatic TB cases in Thailand. Strategy 2 is more effective due to the combined impact of strengthening all three interventions. Increasing active TB treatment coverage ensures more rapid diagnosis and management of infectious individuals, thereby reducing community transmission. At the same time, enhanced preventive therapy helps eliminate the reservoir of potential future cases, preventing progression to active disease. Furthermore, boosting vaccination coverage to 95% provides robust population-level immunity, offering protection to uninfected individuals and contributing to herd immunity.

Strategy 3 involves an implementation of three key TB control interventions, with active TB treatment coverage increased to 90% (0.90), preventive therapy to 70% (0.70), and vaccination coverage to 97% (0.97). As demonstrated in Table 3 and Figure 9, this strategy yields the most significant reduction in both symptomatic and asymptomatic TB cases. A 90% active TB treatment rate ensures that the majority of infectious cases are rapidly diagnosed and treated, substantially limiting onward transmission. Simultaneously, increasing preventive therapy coverage to 70% reduces the pool of latent carriers at risk of progressing to active disease, thereby cutting off a critical source of future infections. The high vaccination rate of 97% contributes to population-level immunity, reducing susceptibility among uninfected individuals and reinforcing herd protection. These findings suggest that high investment in integrated interventions can lead to a significant and sustained decline in TB burden, offering valuable guidance for health policy and TB control efforts in Thailand.

Finally, scaling up all interventions to full coverage over 15 years represents the most intensive TB control effort. Model results show this approach achieves the greatest impact, reducing both symptomatic and asymptomatic cases to near-elimination levels in Thailand. Prompt treatment of active TB eliminates the infectious pool, latent TB treatment removes the hidden reservoir, and universal vaccination lowers overall susceptibility.

The synergistic effect of this fully integrated, high-coverage strategy not only controls the spread of TB but also aligns closely with the World Health Organization’s End TB Strategy goals. However, such an intensive approach requires significant financial, logistical, and human resources. In real-world settings, achieving and sustaining 100% coverage may present practical challenges. Therefore, while this strategy represents the optimal path toward TB elimination, its feasibility is contingent on sustained political commitment and funding. If resource constraints exist, other scenarios—such as the modest investment strategies 2 or 3 (as shown in Table 3)—still offer impactful alternatives and can be considered as phased approaches toward long-term elimination goals.

## 4. Discussion and Conclusions

Tuberculosis (TB) continues to pose a significant public health challenge globally, including in Thailand. Despite progress in recent years, the transmission dynamics and epidemiological patterns of TB within the Thai context remain unclear. This limits the ability to fully evaluate the effectiveness of ongoing interventions or to design context-specific strategies. In response to the persistent burden of TB, the Thai government has implemented a series of national intervention programs aimed at eradicating the disease. Key initiatives have encompassed the provision of free diagnostic and treatment services, Bacillus Calmette–Guérin (BCG) vaccination, management of both active and latent tuberculosis infections, strengthened case detection and reporting mechanisms, and the formulation of clinical and public health guidelines in alignment with international standards.

While these efforts have led to notable improvements in TB control—reflected in declining incidence and mortality rates in many regions—significant challenges persist. Critical gaps remain in case detection, particularly among asymptomatic individuals, and in ensuring continuity of care for those undergoing treatment. Furthermore, delays in accessing timely diagnosis and care, suboptimal vaccination coverage in specific populations, poor treatment adherence, and the growing threat of drug-resistant TB strains continue to undermine the overall effectiveness of control strategies.

In this study, we developed and analyzed a compartmental mathematical model of TB transmission dynamics, explicitly incorporating the effects of vaccination to better understand the epidemiological landscape in Thailand. The model was structured to capture key aspects of TB progression, including latent infection, active disease, diagnosis, treatment, vaccination, and immunization strategies, and estimated the basic reproduction number ($R_{0}$).

To validate and ensure the empirical relevance of our model, we performed calibration using reported TB incidence data from Thai government surveillance sources. Model parameters were either derived from data, the literature, or model fitting. In addition, we conducted a comprehensive sensitivity analysis of the basic reproduction number with respect to key epidemiological and programmatic parameters. The results demonstrated that TB transmission rate, along with the efficacy and coverage of treatment for both latent and active TB, had the greatest influence on $R_{0}$. These findings suggest that targeted efforts to reduce transmission and scale up effective treatment interventions—particularly among latent TB carriers—could significantly lower the reproduction number.

Moreover, this study examined context-specific TB intervention strategies in Thailand by evaluating the projected impact of three key approaches: expanding treatment coverage for latent TB infections (LTBI), enhancing treatment rates for active TB cases, and increasing overall vaccination coverage.

These interventions were selected based on their operational relevance and their capacity to address persistent challenges in the Thai TB program. Using our calibrated compartmental TB model—previously validated with national TB incidence data—we simulated the implementation of these strategies over a 15-year horizon. Our results indicate that increasing treatment coverage for latent tuberculosis (TB) infections had the greatest impact on reducing both asymptomatic and symptomatic TB cases in Thailand. This finding aligns with previous modeling studies and empirical analyses in similar epidemiological contexts [16], which highlight the substantial role of latent TB reservoirs in sustaining long-term transmission.

Latent TB infection, which represents a large pool of individuals harboring *Mycobacterium tuberculosis* (*MTB)* without clinical symptoms, presents a critical challenge for TB control. Without intervention, a proportion of these individuals will progress to active TB over time, particularly under conditions of immunosuppression, poor living conditions, or comorbidities. Identifying and treating latent infections is therefore essential for interrupting future transmission. By preventing the reactivation of latent infections, this approach substantially reduces the incidence of new symptomatic TB cases and curbs overall transmission within the population.

The second most effective intervention was expanding treatment access and adherence among individuals with active TB. While this strategy directly targets the most infectious individuals, reducing their capacity to transmit the disease to others, it is comparatively less effective than preventive therapy in curbing long-term transmission. This is primarily because it does not address the large, often undiagnosed, pool of latent infections that continue to fuel the epidemic over time. Nonetheless, prompt and effective treatment of active TB is vital for reducing immediate incidence and mortality, as well as limiting the development of drug resistance.

Improving vaccination coverage, particularly with the BCG vaccine, was the third most effective strategy. While BCG offers moderate protection against severe TB in children, its efficacy against adult pulmonary TB is variable and declines over time. Therefore, its standalone impact on transmission is limited compared to treatment-based interventions. However, when combined with expanded case detection and treatment, improved vaccination coverage can strengthen population-level immunity and support long-term TB control.

Overall, our findings highlight the importance of a comprehensive TB control strategy that integrates treatment for both latent and active cases, and continued investment in vaccination efforts. These results provide actionable insights for policymakers and public health officials seeking to allocate resources and maximize the impact of TB control programs.

Our analysis demonstrates the substantial epidemiological benefits of combining multiple TB interventions. We compared two strategies: a modest investment approach (Strategy 2) and a comprehensive, high-investment strategy. Strategy 2—with 75% coverage for latent TB treatment, 50% for active TB treatment, and 95% vaccination—significantly reduced both asymptomatic and symptomatic cases. However, full eradication required the high-investment approach, characterized by 100% coverage across all interventions, which under model assumptions eliminated transmission entirely. These findings align with prior research emphasizing that TB elimination in high-burden settings demands extensive coverage and long-term health system commitment.

Importantly, our results highlight the superior effectiveness of combined, multi-component interventions compared to single-policy approaches. Models integrating latent and active TB treatment with robust vaccination consistently outperformed those implementing individual measures alone. This aligns with findings from other infectious disease modeling studies, which show that synergistic interventions can produce nonlinear reductions in disease burden, improv [15,16,18].

In conclusion, this study contributes to the TB modeling literature in Thailand by integrating intervention-specific parameters with sensitivity analysis to inform strategic decision-making. This approach helps identify high-impact interventions, capture nonlinear or delayed responses, and assess the sensitivity of outcomes to changes in key parameters. Additionally, our analysis supports long-term planning and policy decision-making by projecting future trends and illustrating trade-offs between competing strategies. Overall, it enhances the robustness and practical relevance of TB modeling for public health decision-making. The framework can be adapted to other high-burden settings and highlights the importance of combining improved data systems with targeted public health interventions to accelerate TB control efforts.

Several limitations of this study should be acknowledged. First, the model assumes a homogeneously mixed population, overlooking heterogeneity in age, health status, behavior, and socio-economic conditions that influence TB transmission and intervention outcomes. Incorporating population structure in future models could enhance predictive accuracy and policy relevance. Second, the model does not account for acquired or partial immunity. While this may be reasonable in settings with moderate TB transmission, it limits applicability in high-burden areas where immunity significantly affects disease dynamics. Third, several parameters were estimated from reported TB incidence data, which may be affected by under-reporting and misclassification due to limited diagnostic capacity and access to care in Thailand. These data limitations may bias parameter estimates and model outputs. Improved surveillance and diagnostic systems are critical for strengthening model reliability. Finally, the model assumes a constant total population, disregarding demographic changes, migration, and TB-related mortality. This simplification may limit the model’s ability to reflect long-term trends. Despite these limitations, our findings provide useful insights into potential intervention impact and can support evidence-based decision-making in TB control efforts. Future work should incorporate dynamic population structures to better inform public health strategies in Thailand.

## Figures and Tables

**Table 1:** Model parameter values.

Parameters	Description	Values	Units	References
N	Populations in Thailand, 2021	72,000,000	Individuals	[12]
μ	Birth/mortality rate	$\frac{1}{79.27}$	$Year^{-1}$	[12]
β	Transmission rate	$2.8\times 10^{-7}$ $\left(95\%\;CI\;2.3\times 10^{-7},3.3\times 10^{-7}\right)$	$Year^{-1}$	Fitted
ω	The pace of advancement toward the asymptomatic carriage	0.1 (95% CI 0.08,0.119)	$Year^{-1}$	[13]
κ	Rate of progression to symptomatic infection	0.5 (95% CI 0.40,0.59)	$Year^{-1}$	[13]
ρ	Percentage of newly reported cases with symptoms	0.03 (95% CI 0.024,0.035)	$Year^{-1}$	[13]
α	Rate of progression	0.26 (95% CI 0.209,0.311)	$Year^{-1}$	Fitted
ψ	The rate at which latency is recovered	0.23 (95% CI 0.185,0.275)	$Year^{-1}$	Assumed
$\delta_{1}$	The rate at which asymptomatic carriage recovers	0.20 (95% CI 0.161,0.239)	$Year^{-1}$	[14]
$\delta_{2}$	The recovery rate from symptomatic infection	0.205 (95% CI 0.165,0.245)	$Year^{-1}$	[14]
η	Relative infectious of carriers compared to symptomatically infected hosts	0.0006 (95% CI 0.00048,0.00072)	$Year^{-1}$	Fitted
τ	Treatment rate of symptomatic infection	0.60 (95% CI 0.482,0.717)	$Year^{-1}$	[11]
γ	Recovery rate from vaccinated population	0.24 (95% CI 0.193,0.287)	$Year^{-1}$	Assumed
$\sigma_{1}$	Vaccination rate	0.90 (95% CI 0.724,1.076)	$Year^{-1}$	Fitted
$\sigma_{2}$	Progression rate from vaccinated to susceptible	0.20 (95% CI 0.161,0.239)	$Year^{-1}$	Assumed
ϕ	Rate of loss of immunity	0.23 (95% CI 0.185,0.275)	$Year^{-1}$	Assumed
$\mu_{i}$	Disease-related death rate	0.02 (95% CI 0.016,0.024)	$Year^{-1}$	Assumed

**Table 2:** Hypothetical single-intervention strategy implemented in our proposed model for asymptomatic and symptomatic TB control in Thailand for the period 2024–2039.

Parameter	Parameter Value	Estimated Annual Asymptomatic TB Cases $\left(\times 10^{5}\right)$	Reduction from Baseline $\left(\times 10^{5}\right)$	Estimated Annual Symptomatic TB Cases $\left(\times 10^{5}\right)$	Reduction from Baseline $\left(\times 10^{3}\right)$
Active TB treatment (τ)	Baseline	10.67	0.00	4.58	0.00
	0.65	7.97	2.70	3.13	1.45
	0.75	6.61	4.06	2.45	2.13
	0.90	5.03	5.64	1.72	2.86
	1.00	4.22	6.45	1.38	3.20
Preventive therapy (ψ)	Baseline	10.67	0.00	4.58	0.00
	0.30	4.57	6.10	2.02	2.56
	0.50	1.32	9.35	0.61	3.97
	0.70	0.44	10.23	0.22	4.36
	1.00	0.11	10.56	0.05	4.53
Symptomatic reported cases (ρ)	Baseline	10.67	0.00	4.58	0.00
	0.50	9.95	0.72	9.27	−4.69
	0.75	7.05	0.85	12.60	−8.02
	0.85	5.28	3.62	14.05	−9.47
	1	1.94	8.73	16.23	−11.65
$\mathrm{Vaccination}\;(\sigma_{1})$	Baseline	10.67	0.00	4.58	0.00
	0.92	10.18	0.49	4.38	0.20
	0.95	9.48	1.19	4.08	0.50
	0.97	9.05	1.62	3.91	0.67
	1.00	8.44	2.23	3.65	0.93

**Table 3:** Hypothetical multiple intervention strategy implemented in our proposed model for asymptomatic and symptomatic TB control in Thailand for the period 2024–2039.

Scenario	Parameter Changed	Parameter Value	Etimated Annual Asymptomatic TB Cases $\left(\times 10^{5}\right)$	Reduction from Baseline $\left(\times 10^{5}\right)$	Estimated Annual Asymptomatic TB Cases $\left(\times 10^{5}\right)$	Reduction from Baseline $\left(\times 10^{3}\right)$
Baseline	τ	0.50	10.67	0.00	4.58	0.00
	ψ	0.17				
	$\sigma_{1}$	0.90				
Modest investment 1	τ	0.65	3.04	7.63	1.23	3.35
	ψ	0.30				
	$\sigma_{1}$	0.92				
Modest investment 2	τ	0.75	0.57	10.10	0.23	4.35
	ψ	0.50				
	$\sigma_{1}$	0.95				
Modest investment 3	τ	0.90	0.11	10.56	0.04	4.54
	ψ	0.70				
	$\sigma_{1}$	0.97				
Strong investment	τ	1.00	0.02	10.65	0.007	4.57
	ψ	1.00				
	$\sigma_{1}$	1.00				

## Data Availability

Data are contained within the article.

## Abbreviations

The following abbreviations are used in this manuscript:

TB	Tuberculosis
LTBI	Tuberculosis Infection
WHO	World Health Organization
DOTS	Directly Observed Treatment Shortcourse
R_0_	Basic Reproduction Number
S(t)	Susceptible Individuals
V(t)	Vaccinated Individuals
L(t)	Latent Individuals
A(t)	Asymptomatic Infectious Individuals
I(t)	Symptomatic Infectious Individuals
R(t)	Recovered Individuals
BCG	Bacillus Calmette–Guerin
NTP	National Tuberculosis Control Program
PRCC	Partial Rank Correlation Coefficient
LHS	Latin Hypercube Sampling
